# Anti-plaque effect of a surface pre-reacted glass-ionomer-based prophylactic paste under cariogenic challenge: a randomized *in situ* study

**DOI:** 10.1007/s00784-026-07249-0

**Published:** 2026-11-06

**Authors:** ACC Gentile, LY Arima, PS Ferreira, RM Garcia, T Scaramucci, HM Honório, D Rios, M Bönecker

**Affiliations:** 1 School of Dentistry, Department of Orthodontics and Paediatric Dentistry, University of São Paulo, Avenida Professor Lineu Prestes, São Paulo, 2227, CEP: 05508-000 Brazil; 2 School of Dentistry, Department of Restorative Dentistry, University of São Paulo, São Paulo, Brazil; 3 Department of Pediatric Dentistry, Orthodontics and Community Health, Bauru School of Dentistry, University of São Paulo, São Paulo, Bauru Brazil

**Keywords:** Dental enamel, In situ, Cariogenic challenge, Anti-plaque, Bioactive dental materials, Ion-releasing materials

## Abstract

**Objectives:**

To evaluate the anti-plaque effect of a prophylactic paste containing Surface Pre-Reacted Glass-ionomer (S-PRG) fillers under cariogenic challenge using a randomized in situ model.

**Materials and methods:**

This randomized, double-blind study involved 18 volunteers; both the participants and the outcome assessor were blinded to the prophylactic agent allocation. The prophylactic agents were the S-PRG-based Pro-Care Gel (Shofu Inc.; test), Herjos (Vigodent; conventional comparator), and pumice and water (negative control). The other factors were cariogenic challenge (presence or absence) and time of plaque accumulation (24 h and 7 days). During 8 days, volunteers wore intraoral appliances with enamel blocks divided into 2 rows (split-mouth design), in one of them a cariogenic challenge was applied (20% sucrose, 8x/day). Prophylaxis was performed at baseline and again after the 24-hour assessment. The three agent phases were separated by 7-day washout periods. Plaque accumulation was evaluated by quantitative digital image analysis of plaque-covered area (mm²) and adapted Simplified Oral Hygiene Index (OHI-S) scores.

**Results:**

Three-way repeated-measures ANOVA showed significant main effects of Time, Cariogenic Challenge, and Prophylactic Agent (all *p* < 0.001). A significant Time × Prophylactic Agent interaction (*p* = 0.022) indicated different temporal patterns among agents. Plaque accumulation increased from 24 h to 7 days in all groups, while Pro-Care Gel showed the lowest plaque-covered area at both evaluations; the OHI-S analysis showed the same overall group/time pattern.

**Conclusions:**

Under the conditions of this short-term in situ study, the tested S-PRG-based prophylactic paste (Pro-Care Gel) resulted in lower stainable plaque accumulation than the tested conventional paste (Herjos) and pumice/water. These findings are specific to the products and outcomes evaluated.

**Clinical relevance:**

S-PRG prophylactic paste exhibits a sustained anti-plaque effect, markedly reducing dental plaque accumulation relative to the two comparators during the 7-day evaluation period. This strategy could particularly be effective for high-caries-risk patients, as the material’s efficacy is not compromised by frequent sucrose exposure.

## Introduction

Current strategies for dental plaque management rely heavily on mechanical removal; however, for high-risk patients, this approach might be insufficient. Consequently, bioactive materials have gained increasing relevance in contemporary dentistry due to their ability to interact with the oral environment and contribute to dental plaque control beyond conventional mechanical or chemical approaches [1]. Bioactivity encompasses complex biochemical and physiological interactions, including the controlled release of biologically active ions that can influence mineral balance, salivary interactions and bacterial behavior [2, 3]. Accordingly, prophylactic materials may benefit from incorporating sustained bioactive effects that go beyond remineralization, actively modulating surface–bacteria interactions and plaque formation.

Among the available bioactive technologies, Pre-Reacted Glass ionomer (PRG) technology has emerged as a promising approach. PRG technology, which is marketed under the trade name Giomer, is derived from a filler of the same designation. These fillers present a unique multilayered structure, with a silica-modified surface layer on the outside, an ion-exchange layer in the middle, and a multifunctional fluoro-boro-alumino-silicate glass core at the center [3]. This structure enables sustained ion exchange when exposed to aqueous environments. Surface Pre-Reacted Glass ionomer (S-PRG) fillers release fluoride (F⁻) as well as strontium (Sr²⁺), borate (BO₃³⁻), sodium (Na⁺), silicate (SiO₃²⁻), and aluminum (Al³⁺) ions, often at higher concentrations than those observed in conventional dental materials [4]. These ions have been associated with multiple beneficial effects, including acid buffering, enhancement of mineral resistance, and modulation of bacterial adhesion, suggesting a potential role in controlling dental plaque accumulation [3–4].

Previous in vitro studies have reported reduced bacterial adhesion and plaque accumulation on surfaces containing S-PRG particles, initially indicating an inhibitory effect primarily on single-species models, such as *Streptococcus mutans* [5–8]. However, these simplified laboratory models fail to simulate the microbiological and structural complexity of the natural dental biofilm. To address this limitation, more robust investigations have recently evaluated the antimicrobial efficacy of S-PRG using microcosm biofilm models, which better represent the ecological diversity of the oral cavity [9]. While microcosm studies provide valuable insights, they remain in vitro approaches that do not fully replicate the dynamic conditions of the oral environment, such as the acquired pellicle formation and continuous salivary flow. Therefore, taking a step further toward in vivo conditions through in situ models is crucial to comprehensively understand the clinical effects of S-PRG-based materials on plaque formation [10].

While prophylactic pastes containing S-PRG fillers have demonstrated improvements in enamel surface hardness and smoothness [11–12], no in situ study has yet assessed the ability of this S-PRG–based paste to control dental plaque accumulation in a model simulating patients at elevated caries risk. Therefore, the aim of this randomized in situ study was to assess the anti-plaque effect of a prophylactic paste containing PRG technology on human enamel specimens under cariogenic challenge. The null hypothesis was that there would be no significant difference in plaque accumulation between the S-PRG-based paste and either pumice and water or conventional prophylactic pastes, under a cariogenic challenge.

## Materials and methods

### Experimental design

The Research Ethics Committee of the local university approved this project (Certificate of Presentation for Ethical Consideration - CAAE: 79258224.0.0000.0075). This in situ study followed a randomized, double-blind, crossover, and split-mouth design. Participants and the outcome assessor were blinded to the prophylactic agent allocation. Dental plaque accumulation on enamel specimens was evaluated according to three factors: (1) Prophylactic Agent at three levels: test prophylactic paste (S-PRG-based paste; Pro-Care Gel, Shofu Inc.); conventional comparator (non-S-PRG prophylactic paste; Herjos, Vigodent, Rio de Janeiro, Brazil); and negative control (pumice and water); (2) Cariogenic Challenge (presence or absence); and (3) Time of plaque accumulation (24 h and 7 days).

The protocol comprised three experimental phases of 8 days each, separated by a 7-day washout period. Participants wore removable maxillary acrylic intraoral appliances containing four niches (5 × 5 mm) with enamel specimens. A split-mouth design was applied for the cariogenic challenge, while the prophylactic agents were tested in a crossover manner. An in situ model was selected because it exposes treated human enamel to acquired pellicle formation, saliva, and the complex oral environment while maintaining experimental control over the substrate, prophylactic procedure, and sucrose exposure. The 24-hour time point was selected to characterize early plaque recolonization after prophylaxis, whereas 7 days was selected to assess whether between-agent differences persisted during a longer plaque-accumulation period while keeping each crossover phase feasible for repeated participant wear.

### Subjects

The sample size was calculated from an unpublished pilot in situ study involving six volunteers, conducted using the same methodology as the definitive study, including the intraoral appliance design, prophylaxis protocol, cariogenic challenge, evaluation periods, and plaque assessment procedures. Using an α-error of 5%, a β-error of 20% (80% power), a standard deviation of 2.77 mm², and an estimated minimum detectable difference of 4 mm² in plaque-covered area, a sample of 18 volunteers was required. Eighteen healthy volunteers (aged 21–35 years) with no active dental disease, no gingival inflammation, and no relevant medical history were recruited. Screening was conducted at the Postgraduate Dental Clinic. Exclusion criteria comprised: allergy to any dentifrice ingredient, pregnancy or lactation, antibiotic or anti-inflammatory usage in the preceding month, use of fixed orthodontic appliances, presence of oral pathology, xerostomia/reduced salivary flow, or professional topical fluoride application within the last 2 months. Written informed consent was obtained from all participants.

### Specimens

Human enamel was selected as the experimental substrate because professional prophylaxis is clinically performed directly on enamel surfaces, and the use of intact human enamel provides a biologically and topographically relevant surface for studying plaque recolonization. Enamel specimens were prepared from extracted human permanent molars (*n* = 75) obtained from the local Human Teeth Bank. Teeth presenting cracks, caries lesions, defects, or wear were excluded. After cleaning, teeth were stored in distilled water at 4 °C. Coronal enamel was sectioned under water cooling using a precision diamond saw (IsoMet 1000, Buehler, Lake Bluff, Illinois, United States of America) to obtain square specimens. To achieve a uniform thickness of approximately 2 mm, only the dentin surface was ground flat using silicon carbide paper under water cooling. The outer enamel surface remained intact (natural) and was not subjected to any grinding or polishing procedures to preserve its original topography. The final specimens (4 × 4 × 2 mm) were sterilized by gamma irradiation (25 kGy) prior to the experimental procedures.

### Intraoral appliances and in situ protocol

**Fig. 1:**
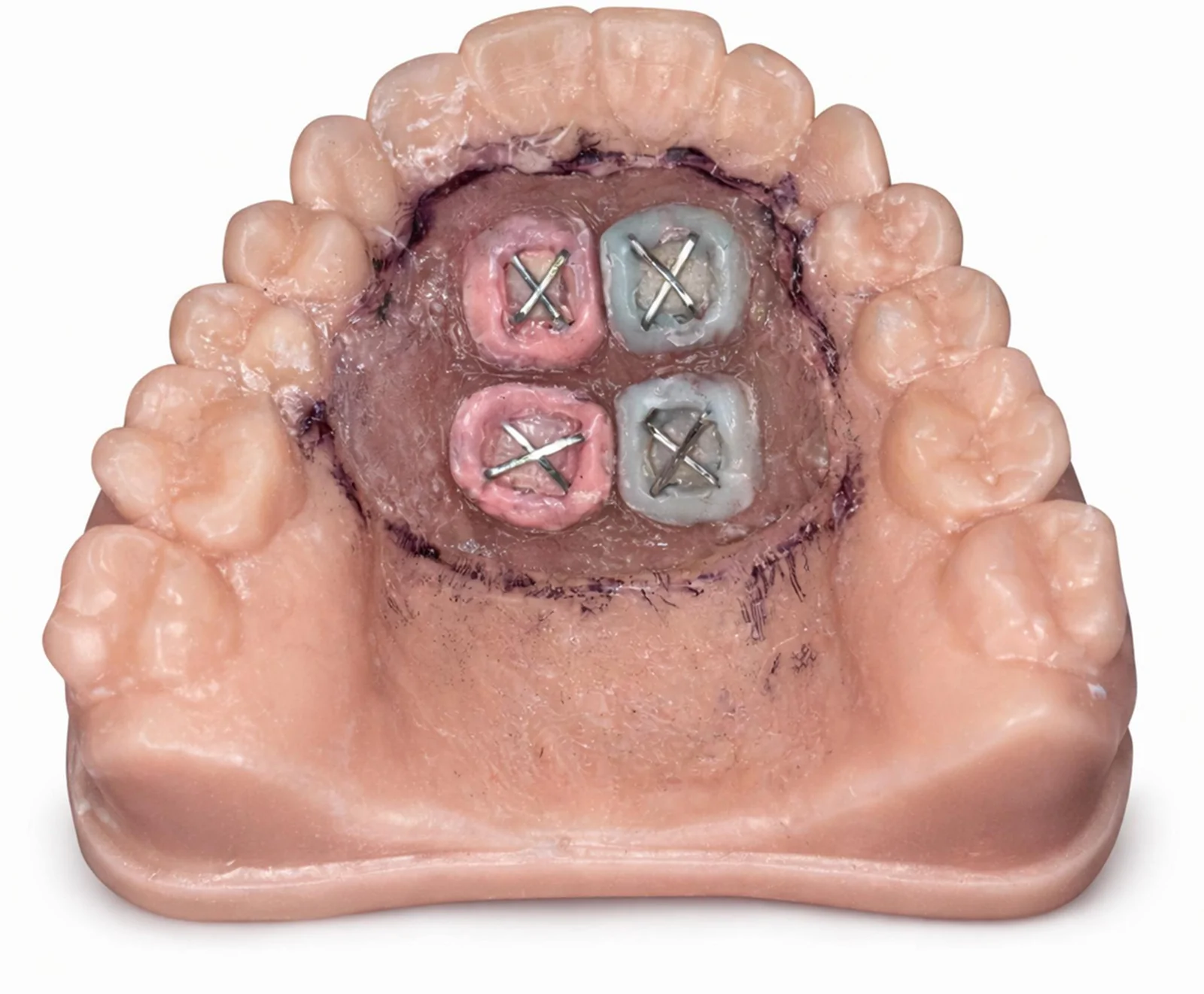
Removable maxillary acrylic intraoral appliance on a three-dimensional printed model, showing the bilateral specimen niches and the X-shaped protective wire grid positioned over the enamel specimens

Intraoral scans (TRIOS, 3Shape, Copenhagen, Denmark) were obtained to generate 3D-printed models for fabrication of customized removable maxillary acrylic appliances using autopolymerizing acrylic resin. Intraoral retention was provided by close adaptation of the appliance to the palatal surface, producing a vacuum/suction effect. Each appliance contained two vertical rows with bilateral cavities to house four enamel specimens (two per side/row), fixed with modeling wax. To model repeated cariogenic exposure, two specimens on one randomized side were subjected to a 20% sucrose solution eight times daily [13]. Distinct wax colors (pink for cariogenic challenge and blue for control) were used to guide the volunteers, while the left-right positioning of the challenged specimens was randomized across participants. An X-shaped protective grid (0.7-mm stainless-steel wire) was placed over each specimen to reduce direct mechanical disturbance of accumulating plaque by the tongue (Fig. 1). The same grid configuration was used for all specimens and all experimental conditions so that any grid-related influence on local plaque retention would be applied systematically across groups. Participants received standardized oral hygiene kits (soft toothbrush and fluoridated toothpaste, 1450 ppm F) and written instructions. A computerized random number generator (Excel) was used to determine the sequence of prophylactic agents and the side of the cariogenic challenge for each volunteer.

At the beginning of each phase, participants wore the appliance for 2 h to allow for initial acquired pellicle formation. This 2-hour period was included within the first 24-hour cycle of the experiment. Subsequently, the appliances were temporarily removed from the mouth, and the baseline prophylaxis was performed extraorally by a single trained operator. A standardized amount of 0.7 g of the respective prophylactic agent was applied using a soft Robinson brush mounted on a low-speed handpiece for 10 s per specimen, applying light force. For the pumice group, a standardized paste was prepared using a 1:1 mass/volume ratio of pumice powder and distilled water.

**Fig. 2:**
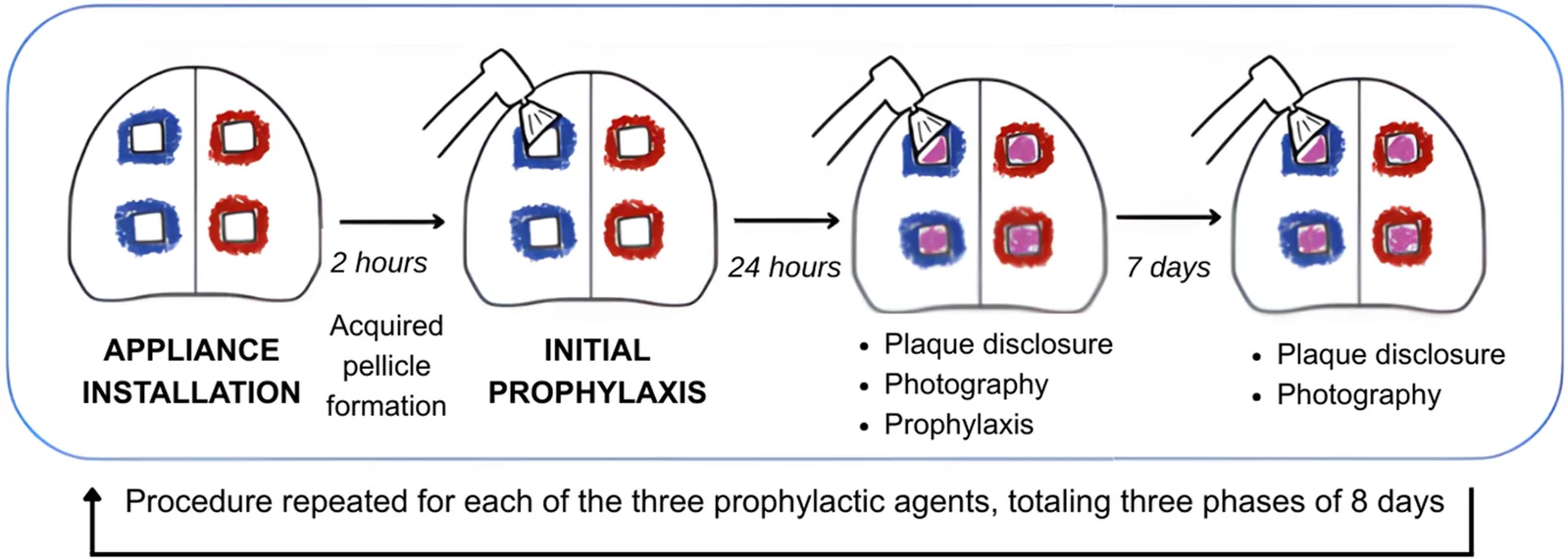
Schematic representation of the experimental procedures

Immediately after the baseline prophylaxis, the appliances were reinserted into the participants’ mouths. At the 24-hour mark (Day 1), the appliances were removed for the first data collection (plaque disclosure and imaging). Following this assessment, a second prophylaxis was performed identically to the baseline procedure. The appliances were then immediately reinserted into the mouth until the 7-day mark (Day 8), when the final data collection was conducted. After this 8-day period and a subsequent 7-day washout, the crossover procedure was repeated with a new appliance and the next prophylactic agent in the sequence (Fig. 2).

### Analysis of dental plaque

Plaque accumulation was assessed using two methods: quantitative plaque coverage area and clinical plaque scores. Plaque disclosure was performed by applying two drops of a disclosing solution (Viplak, AF do Brasil, Londrina, Brazil) to each specimen. The solution remained on the surface for 10 s before the data collection.

#### Quantitative assessment

Standardized photographs were captured using a smartphone fixed on a support to ensure constant distance, angulation, and lighting conditions. Images were analyzed using ImageJ software (National Institutes of Health, Bethesda, Maryland, United States of America) [14]. The total visible specimen area and the area covered by stained plaque were measured; plaque-covered area, expressed in mm², was used as the quantitative outcome. The analyst was blinded to the three groups.

#### Simplified oral hygiene index (OHI-S) scores

An adapted Simplified Oral Hygiene Index (OHI-S) [15] was used. Specimens were scored by direct observation immediately after plaque disclosure, before photographic analysis, by the same blinded outcome assessor who performed the quantitative image analysis. In doubtful cases, a specialist examiner was consulted to support scoring. Examiner reproducibility was high, with an intra-examiner Kappa of 1.00 and an inter-examiner Kappa of 0.90. Specimens were scored from 0 to 3: (0) no plaque; (1) plaque covering ≤ 1/3; (2) plaque covering > 1/3 but ≤ 2/3; and (3) plaque covering > 2/3 of the exposed enamel surface.

### Statistical analysis

Statistical analysis was performed using STATISTICA software version 10.0 (StatSoft Inc., Tulsa, Oklahoma, United States of America). The assumptions of normality and homogeneity of variance were verified using the Shapiro-Wilk test. For both outcomes (plaque-covered area [mm2] and OHI-S scores [15]), data were analyzed using a three-way repeated-measures analysis of variance (ANOVA) considering the factors: Prophylactic Agent (S-PRG-based paste, Non-S-PRG conventional prophylactic paste, Pumice and water), Cariogenic Challenge (With, Without), and Time (24 h, 7 days). Tukey’s post hoc test was used for multiple comparisons when significant differences were detected. The significance level was set at 5%. To determine the magnitude of the observed effects and their clinical significance, the partial eta-squared was calculated for all main effects and interactions. According to Cohen’s benchmarks, values of 0.01, 0.06, and 0.14 were interpreted as small, medium, and large effect sizes, respectively [16].

## Results

### Quantitative results

The quantitative assessment of plaque-covered area is summarized in Table 1. The three-way repeated-measures ANOVA revealed significant main effects of Time (*p* < 0.001; partial eta-squared = 0.78), Cariogenic Challenge (*p* < 0.001; partial eta-squared = 0.83), and Prophylactic Agent (*p* < 0.001; partial eta-squared = 0.78). Furthermore, a significant interaction was detected between Time and Prophylactic Agent (*p* = 0.022; partial eta-squared = 0.23), indicating that the progression of plaque accumulation differed among the interventions over time. At 24 h, the S-PRG-based paste demonstrated significantly lower plaque-covered area compared with both the conventional prophylactic paste and the pumice group, which did not differ from each other. At 7 days, all groups differed significantly; the S-PRG-based paste maintained the lowest values, the conventional paste showed intermediate values, and the pumice group exhibited the highest plaque-covered area. Within each prophylactic agent group, plaque accumulation was significantly higher at 7 days than at 24 h. The Prophylactic Agent × Cariogenic Challenge interaction was not significant (*p* = 0.450; partial eta-squared = 0.016). The Time × Cariogenic Challenge interaction was also not significant (*p* = 0.909; partial eta-squared < 0.001), nor was the Time × Prophylactic Agent × Cariogenic Challenge interaction (*p* = 0.895; partial eta-squared = 0.002). Overall, sucrose-exposed specimens showed greater plaque accumulation, consistent with the significant main effect of Cariogenic Challenge.

**Table 1:** Quantitative plaque coverage (mm²) according to prophylactic agent, cariogenic challenge, and time

Prophylactic agent	Cariogenic challenge	24 h mean (SD)	7 days mean (SD)
S-PRG Prophylactic Paste	Without	1.56 (0.88) ^a, A,ф^	3.97 (2.49) ^a, A,ᴪ^
	With	2.27 (0.89) ^b, A,ф^	5.02 (2.68) ^b, A,ᴪ^
Conventional Prophylactic Paste	Without	6.09 (2.72) ^a, B,ф^	8.76 (2.60) ^a, B,ᴪ^
	With	8.00 (3.14) ^b, B,ф^	10.88 (3.06) ^b, B,ᴪ^
Pumice and Water	Without	6.25 (2.86) ^a, B,ф^	10.90 (2.39) ^a, C,ᴪ^
	With	7.92 (2.96) ^b, B,ф^	12.25 (1.65) ^b, C,ᴪ^

*Data are presented as mean and standard deviation (SD). Different lowercase superscript letters indicate statistically significant differences in cariogenic challenge (with or without) within the same prophylactic agent and within the same evaluation period. Different uppercase superscript letters indicate statistically significant differences among prophylactic agents (S-PRG Prophylactic Paste, Conventional Prophylactic Paste and Pumice and Water) within each type of cariogenic challenge and within each evaluation time point. Different superscript symbols indicate statistically significant differences between the evaluation times (24 h and 7 days) within each type of cariogenic challenge and within each prophylactic agent. (Three-way repeated-measures ANOVA followed by Tukey’s post hoc test, p < 0.05).

### Simplified oral hygiene index (OHI-S) scores

The analysis of the OHI-S scores is presented in Table 2. Consistent with the quantitative findings, the ANOVA showed significant main effects of Time (*p* < 0.001; partial eta-squared = 0.68), Cariogenic Challenge (*p* < 0.001; partial eta-squared = 0.62), and Prophylactic Agent (*p* < 0.001; partial eta-squared = 0.76). A significant Time × Prophylactic Agent interaction was also observed for the OHI-S scores (*p* = 0.027; partial eta-squared = 0.16). At the 24-hour evaluation, the S-PRG-based paste group exhibited significantly lower scores than the conventional prophylactic paste and pumice groups. At the 7-day evaluation, the groups exhibited distinct performances: the S-PRG-based paste maintained the lowest scores, followed by the conventional prophylactic paste, while the pumice group reached the highest scores. For all evaluated prophylactic agents, the OHI-S scores were significantly higher at 7 days than at 24 h. The Time × Cariogenic Challenge interaction was not significant (*p* = 0.432; partial eta-squared = 0.006), the Prophylactic Agent × Cariogenic Challenge interaction was not significant (*p* = 0.325; partial eta-squared = 0.022), and the Time × Prophylactic Agent × Cariogenic Challenge interaction was not significant (*p* = 0.827; partial eta-squared = 0.004). Overall, sucrose exposure resulted in higher OHI-S scores, consistent with the significant main effect of Cariogenic Challenge.

**Table 2:** Simplified Oral Hygiene Index (OHI-S) scores according to prophylactic agent, cariogenic challenge, and time

Prophylactic agent	Cariogenic challenge	24 h mean (SD)	7 days mean (SD)
S-PRG Prophylactic Paste	Without	0.83 (0.30) ^a, A,ф^	1.31 (0.55) ^a, A,ᴪ^
	With	0.92 (0.19) ^b, A,ф^	1.39 (0.58) ^b, A,ᴪ^
Conventional Prophylactic Paste	Without	1.58 (0.52) ^a, B,ф^	2.11 (0.61) ^a, B,ᴪ^
	With	2.00 (0.75) ^b, B,ф^	2.42 (0.62) ^b, B,ᴪ^
Pumice and Water	Without	1.61 (0.56) ^a, B,ф^	2.56 (0.42) ^a, C,ᴪ^
	With	1.94 (0.54) ^b, B,ф^	2.69 (0.25) ^b, C,ᴪ^

*Data are presented as mean and standard deviation (SD). Different lowercase superscript letters indicate statistically significant differences in cariogenic challenge (with or without) within the same prophylactic agent and within the same evaluation period. Different uppercase superscript letters indicate statistically significant differences among prophylactic agents (S-PRG Prophylactic Paste, Conventional Prophylactic Paste and Pumice and Water) within each type of cariogenic challenge and within each evaluation time point. Different superscript symbols indicate statistically significant differences between the evaluation times (24 h and 7 days) within each type of cariogenic challenge and within each prophylactic agent. (Three-way repeated-measures ANOVA followed by Tukey’s post hoc test, *p* < 0.05).

## Discussion

To the best of our knowledge, this is the first in situ study to evaluate plaque accumulation after prophylaxis with Pro-Care Gel, the S-PRG-based paste tested in this study. The null hypothesis was rejected because Pro-Care Gel showed lower OHI-S scores and quantitative plaque coverage than the tested conventional paste (Herjos) and pumice/water in the factorial analysis. These findings provide product-specific in situ evidence and are consistent with previous laboratory reports describing reduced biofilm formation or altered bacterial behavior associated with S-PRG-containing materials [5–9]. However, the present results should not be generalized to all S-PRG-based or conventional prophylactic pastes.

The lower plaque coverage observed after treatment with Pro-Care Gel may be related to physicochemical properties previously reported for S-PRG-containing materials, including multi-ion release [3–4]. However, ion release, bacterial viability, bacterial composition, extracellular matrix, and bacterial adhesion were not measured in the present study; therefore, these mechanisms remain hypotheses rather than demonstrated explanations for the observed plaque-area differences. Prior in vitro studies have reported effects of S-PRG eluates or S-PRG-containing materials on bacterial metabolism, biofilm formation, and adhesion [17–21], whereas other ion-releasing bioactive glass systems have not shown inhibition of bacterial adhesion [18]. Accordingly, the present stainable plaque measurements should not be interpreted as direct evidence of bactericidal or anti-adhesive activity. Potential contributions of fluoride, strontium, borate, or other released ions to the observed findings require direct mechanistic confirmation [17, 22–24].

Surface topography is another factor that may influence plaque retention [25–27], and previous studies have reported changes in enamel surface properties after use of S-PRG-containing prophylactic materials [12, 28]. Nevertheless, baseline or post-treatment surface roughness, surface energy, contact angle, and surface chemistry were not measured in the present study. Since the S-PRG-based paste is formulated with non-abrasive ultrafine fillers, the hypothesis is that it promotes a smoother surface topography [28] naturally less conducive to plaque development. Therefore, the increased plaque coverage observed in the control groups after 7 days, confirmed by the significant interaction between Time and Prophylactic Agent, may hypothetically be partially attributed to a rougher surface micro-morphology that facilitated rapid bacterial recolonization, unlike the smoother, chemically protected surface of the S-PRG group. The significant Time × Prophylactic Agent interaction demonstrates that the temporal pattern of plaque accumulation differed among the three tested conditions, but it does not establish the biological mechanism responsible for that difference. The high partial eta-squared values obtained for the Prophylactic Agent (0.78) indicate that the bioactive technology (PRG) explains more than 75% of the variance in plaque reduction, representing a robust clinical effect. Furthermore, the interaction between Time and Prophylactic Agent showed a large effect size (0.23), statistically confirming that the S-PRG group follows a distinct and significantly slower plaque recolonization pattern compared to conventional methods over 7 days relative to the two comparators used in this study.

The experimental model incorporated a repeated 20% sucrose challenge, a well-established approach for increasing cariogenic pressure in in situ biofilm studies [13, 29–30]. The significant main effect of Cariogenic Challenge showed that sucrose exposure was associated with greater stainable plaque accumulation across the experiment, supporting the responsiveness of the model. The split-mouth design for the challenge and crossover design for the prophylactic agents reduced inter-individual variability. In addition, the OHI-S and quantitative plaque-area analyses showed the same overall pattern of group and time effects. This supports consistency between an ordinal visual score and a digital areal measure; however, no formal correlation or agreement analysis between the two methods was performed. They should therefore be regarded as complementary outcome measures rather than quantitatively interchangeable assessments.

Despite the promising findings, some limitations should be acknowledged: (1) The experimental phase of 8 days does not allow for the assessment of long-term cumulative effects or the “ion recharge” capacity of the material over extended periods [31]; (2) As the present study evaluated specific commercially available prophylactic products, the findings should be interpreted within the context of the materials tested and should not be extrapolated to other products with similar compositions or indications; (3) The natural enamel surface was intentionally preserved, so baseline roughness heterogeneity among specimens may have contributed to variability; (4) The X-shaped protective wire grid was used identically across conditions to limit tongue-related mechanical disturbance, but it may have locally influenced plaque-retention conditions. (5) As in other in situ studies, the protocol depended on participant adherence to appliance-wear and sucrose-application instructions, which may introduce compliance-related variability. Finally, future studies should compare multiple commercial products, incorporate microbiological validation and direct characterization of surface/ion-release changes, and extend observation to clinical endpoints.

## Conclusions

Within the limitations of this randomized, double-blind crossover in situ study, the tested S-PRG-based prophylactic paste (Pro-Care Gel) resulted in lower stainable plaque-covered area and OHI-S scores than the tested conventional paste (Herjos) and pumice/water at both evaluation periods. Plaque accumulation increased from 24 h to 7 days for all tested agents, and the significant Time × Prophylactic Agent interaction indicated different temporal patterns among treatments. These short-term findings are specific to the products and outcome measures tested and do not establish microbiological mechanisms, long-term caries prevention, or class-wide efficacy of S-PRG-containing materials.

## Data Availability

All data generated or analyzed during this study are included in this manuscript.
